# Beyond Neddylation Inhibition: X‑ray Structures Reveal Carbonic Anhydrase Isoform Selectivity of Pevonedistat

**DOI:** 10.1021/acsmedchemlett.6c00197

**Published:** 2026-05-27

**Authors:** Chiara Baroni, Marta Ferraroni, Claudiu T. Supuran, Andrea Angeli

**Affiliations:** † Department of Chemistry “Ugo Schiff”, 9300 University of Florence, Via della Lastruccia 3-13, I-50019 Sesto Fiorentino, Florence, Italy; ‡ NEUROFARBA Department, Sezione di Scienze Farmaceutiche, University of Florence, Via Ugo Schiff 6, 50019 Sesto Fiorentino, Florence, Italy

**Keywords:** carbonic anhydrase, metalloenzyme, pevonedistat, tumor, neddylation, sulfamate

## Abstract

Pevonedistat (MLN4924) is a first-in-class inhibitor of the NEDD8-activating enzyme that blocks protein neddylation and exhibits antitumor activity in multiple clinical phases. Here, we report a previously unrecognized and isoform-selective inhibitory profile of pevonedistat against the tumor-associated isoforms human carbonic anhydrase IX and XII (hCA IX and XII). To elucidate the structural basis of this selectivity, X-ray crystal structures were determined for pevonedistat in complex with hCA I, hCA II, and an engineered hCA II variant mimicking hCA XII. These findings provide a mechanistic explanation for the known preferential partitioning of pevonedistat into whole blood via binding to erythrocyte CAs and suggest that CA inhibition may contribute to its antitumor activity in hypoxic tumor microenvironments where hCA IX and XII are overexpressed. This study reveals a dual functional profile for pevonedistat, linking neddylation inhibition with selective targeting of tumor-associated CAs and offers to exploit this synergy in anticancer drug design.

## Graphical abstract

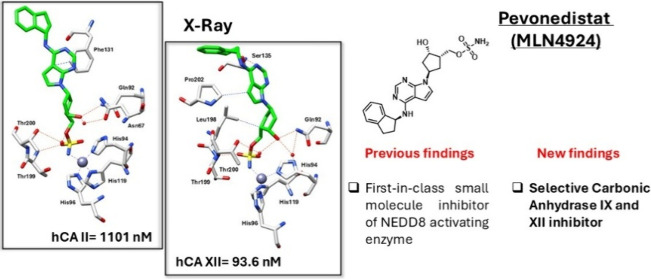

Pevonedistat (also known as MLN4924 or TAK-924) is an adenosine sulfamate analog and the first-in-class small molecule inhibitor of the neural precursor cell expressed developmentally downregulated 8 (NEDD8) activating enzyme (NAE) (Figure 1).^1^ It acts by forming a covalent adduct that mimics the adenylated NEDD8 intermediate, thereby preventing formation of the thioester bond required for downstream transfer steps in the neddylation cascade.^1,2^

**Figure 1:**
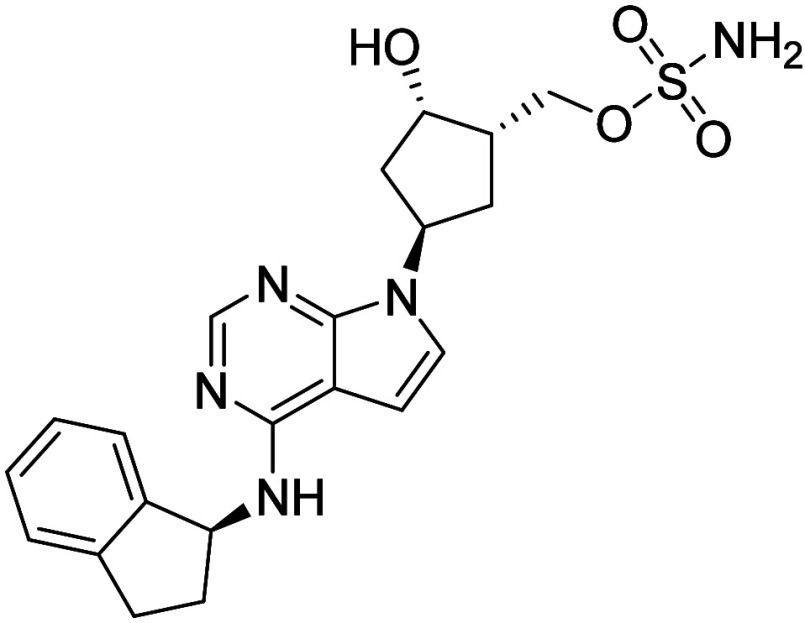
Structure of pevonedistat (MLN4924).

This conjugation pathway, termed neddylation, is mechanistically and structurally analogous to ubiquitination. Indeed, NEDD8 shares significant sequence homology with ubiquitin and functions via a similar mechanism.^1,3,4^ The E1 enzyme responsible for NEDD8 activation, NEDD8-activating enzyme E1 regulatory subunit, forms a heterodimer with Ubiquitin-like modifier-activating enzyme 3 (UBA3). This complex catalyzes ATP-dependent adenylation of NEDD8 to form a NEDD8-AMP intermediate within the nucleotide-binding pocket, followed by transfer to a catalytic cysteine residue to generate a thioester-linked NEDD8–NAE intermediate. Binding of a second NEDD8-AMP molecule induces a conformational state that promotes transfer of the first NEDD8 to the cognate E2 conjugating enzyme.^1^ Approximately 20% of cellular proteins are degraded through the ubiquitin-proteasome system (UPS), largely regulated by Cullin-RING ubiquitin ligase (CRL) E3 ligases. Activation of cullins requires covalent conjugation of NEDD8, making neddylation a critical regulatory step for CRL activity.^5^ Dysregulation of this pathway has been implicated in multiple pathophysiological processes, including cell survival and differentiation, neurodegeneration, and cancer.^6–8^ Dysregulation of neddylation signaling in cancer is well documented, with multiple studies reporting overexpression of NEDD8 and its associated enzymes in tumor tissues.^5^ By inactivating CRL, pevonedistat exhibits pronounced antitumor activity and have advanced through numerous Phase I/II clinical trials for the treatment of both hematological malignancies and solid tumors.^1,9^ It has also been evaluated in Phase III clinical trials in combination with azacitidine for the treatment of Acute Myeloid Leukemia.^10–13^ An important pharmacokinetic observation emerging from these studies is the substantially higher concentration of pevonedistat in whole blood compared with plasma.^14^ This preferential partitioning into red blood cells has been attributed to binding to ubiquitous human carbonic anhydrases (CAs, EC 4.2.1.1) I and II, two highly abundant isoforms present in erythrocytes.^15^ CAs are zinc metalloenzymes that catalyze the reversible hydration of carbon dioxide to bicarbonate and a proton.^16,17^ The binding of pevonedistat to these enzymes provides a plausible explanation for the extensive whole-blood distribution observed in both animal models and humans.^14^ On the other hand, in solid tumors, hypoxic conditions and the resulting acidic microenvironment confer a selective advantage to malignant cells.^18^ To survive under acidic and hypoxic stress, tumor cells upregulate mechanisms that maintain intracellular pH within a slightly alkaline range conducive to proliferation and survival.^19,20^ Among these adaptations is the overexpression of the tumor-associated isoforms hCA IX and XII.^17,20^ Consequently, increasing evidence supports CA inhibition as a promising anticancer strategy.^21–23^ In this context, the presence of a sulfamate moiety in the pevonedistat scaffold may contribute to its antitumor activity. In fact, the sulfamate is a recognized bioisostere of the sulfonamide, the typical zinc binding group of CAs, and is itself a characteristic zinc-binding motif in CA inhibitors. This dual functionality provides a mechanistic rationale for a synergistic effect arising from the combination of neddylation inhibition and CA inhibition in solid tumors. The inhibitory activity of pevonedistat was evaluated against all catalytically active human CA isoforms (hCA I-XIV) and compared with the reference inhibitor acetazolamide (AAZ) and the clinically used sulfamate topiramate, using a stopped-flow CO_2_ hydration assay (Table 1).

**Table 1:** Inhibition Data of All Catalytically Active Human CAs (hCA I–XIV) with Pevonedistat, Topiramate and AAZ Determined by a Stopped-Flow CO_2_ Hydrase Assay

	K_I_ (nM)^a^		
Compd	Pevonedistat	AAZ	Topiramate
**hCA I**	262.6	250.0	250.0
**hCA II**	1101	12.1	10.0
**hCA III**	>10000	>10000	>10000
**hCA IV**	93.7	74.0	4900
**hCA VA**	709.1	63.0	63.0
**hCA VB**	2870	54.0	30.0
**hCA VI**	830.0	11.0	45.0
**hCA VII**	75.8	2.5	0.9
**hCA IX**	59.1	25.7	58.0
**hCA XII**	93.6	5.7	3.8
**hCA XIII**	323.8	17.0	47.0
hCA XIV	1959	41.0	1460

a Mean from 3 different assays, by a stopped flow technique (errors were in the range of ±5–10% of the reported values).

Cytosolic isoforms (hCA I, II, III, VII, and XIII) were inhibited by pevonedistat to varying degrees. Notably, hCA I displayed comparable inhibition to both topiramate and AAZ (K_I_ = 262.6 nM), and none of the tested compounds inhibited hCA III. In contrast, pevonedistat exhibited markedly weaker inhibition against hCA II (K_I_ = 1101 nM), approximately 3 orders of magnitude less potent than AAZ and topiramate. Among the remaining cytosolic isoforms, hCA VII was one of the most effectively inhibited with a K_I_ of 75.8 nM, whereas hCA XIII showed moderate inhibition (K_I_ = 323.8 nM), similar to that observed for hCA I. Regarding mitochondrial isoforms (hCA VA and VB), pevonedistat demonstrated weak inhibitory activity, with K_I_ values in the high nanomolar to low micromolar range (709.1 nM and 2870 nM, respectively). In contrast, both AAZ and topiramate exhibited significantly stronger inhibition in the midnanomolar range. Similarly, hCA VI was inhibited by pevonedistat with a K_I_ of 830 nM, corresponding to an approximately 18-fold and 75-fold reduction in potency compared to topiramate and AAZ, respectively. For the membrane-associated isoform hCA IV, pevonedistat displayed inhibition comparable to AAZ (K_I_ = 93.7 vs 74.0 nM), whereas topiramate was over 50-fold less potent. Importantly, the tumor-associated isoforms hCA IX and XII were effectively inhibited by pevonedistat. In particular, hCA IX showed the strongest inhibition (K_I_ = 59.1 nM), comparable to both AAZ and topiramate, while hCA XII was inhibited with moderate potency (K_I_ = 93.6 nM). Finally, hCA XIV was weakly inhibited by pevonedistat (K_I_ = 1959 nM), similarly to topiramate. Overall, pevonedistat demonstrates a favorable inhibition profile, with potent activity against tumor-associated isoforms (hCA IX and XII) comparable to AAZ and topiramate, while exhibiting reduced activity against off-target isoforms such as hCA I and II. This enhanced selectivity may contribute to its therapeutic efficacy in solid tumors, where these hCAs isoforms are overexpressed.^24–26^

To rationalize the selectivity of pevonedistat toward tumor-associated CA isoforms, X-ray crystal structures were determined for hCA I, hCA II, and an engineered hCA II variant carrying active-site mutations designed to mimic hCA XII, (hCA XII mimic^27^) each in complex with pevonedistat. In all structures, the inhibitor displayed well-defined electron density within the active site (Figure S1), allowing unambiguous modeling of its binding mode. In every adduct, the sulfamate group coordinates the catalytic Zn ion in its deprotonated form,^28^ in a manner analogous to classical sulfonamide inhibitors (Figure 2).^29^ Consistently across the three complexes, one sulfamate oxygen forms a hydrogen bond with the backbone amide of Thr199, a conserved interaction known to stabilize inhibitors of this class within the CA active site (Figure 2).^28–31^ In the hCA I complex, a single van der Waals interaction is observed between Leu198 and the pentacyclic core of the inhibitor (Figure 2A). The adenine moiety forms a hydrogen bond with Pro201 and participates in two water bridge interactions involving Gln92 and His200. Additionally, the phenyl ring of the dihydro-indene tail engages in double π-stacking interactions with the aromatic side chain of Trp5, further stabilizing the inhibitor within the binding pocket (Figure 2A). In the hCA II complex, two water bridges are also present but involved different residues and portions of the inhibitor (Figure 2B). One water molecule bridges Thr200 and sulfamate oxygen, similarly to interactions reported for aliphatic sulfamides.^30,31^ The second water bridge connects the alcohol group of the pentacyclic ring with Asn67; this hydroxyl group also forms a hydrogen bond with Gln92. Only a single van der Waals interaction is observed, between Phe131 and the adenine ring (Figure 2B). A comparison of the hCA I and hCA II complexes reveals a reduced number of favorable interactions in hCA II, consistent with the weaker inhibition observed for hCA II relative to hCA I (K_I_ = 1101 nM vs 262.6 nM). Moving on the hCA XII mimic complex, two water bridge interactions again involve the alcohol group of the pentacyclic ring (Figure 2C). One water bridge connects this group with Gln92 and His94, while the second involves Thr200. This interaction network reorients the cyclopentyl portion of the inhibitor, restoring the van der Waals contact with Leu198 observed in hCA I but absent in hCA II. A key determinant is residue 131. In hCA II, Phe131 sterically constrains the inhibitor tail in a suboptimal orientation that limits contacts (Figure S2). In the hCA XII mimic, replacement of Phe131 with the less bulky Ala131 allows the tail to locate deeper into the hydrophobic pocket.

**Figure 2:**
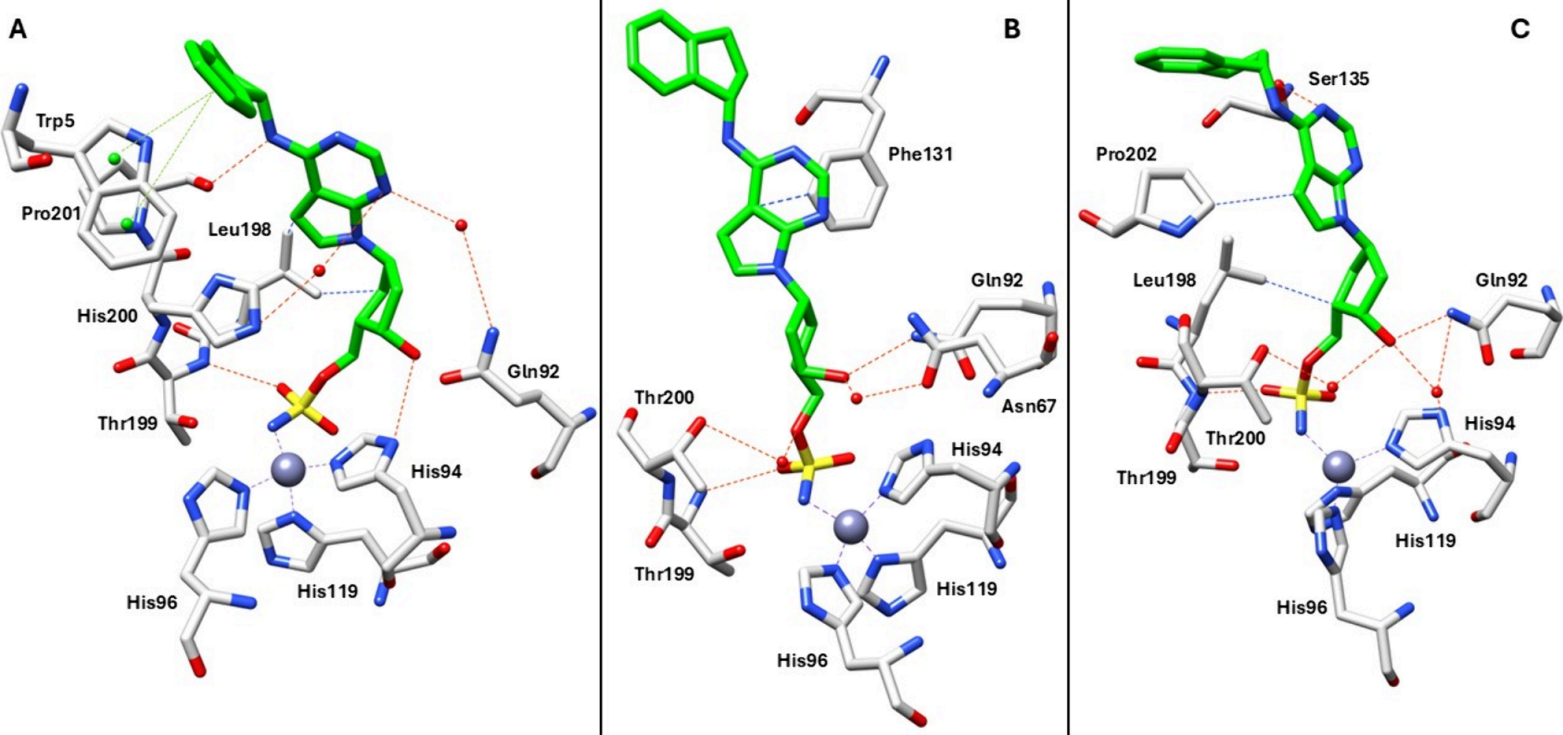
X-ray crystal structure of pevonedistat bound with hCA I (**A**, PDB: 29UD), hCA II (**B**, PDB: 29UH) and hCA XII mimic (**C**, PDB: 29UX). Residues involved in the binding of inhibitor are also shown; the gray sphere represents the zinc ion in the active site of the proteins. The purple dotted lines show the coordination of the zinc ion, van der Waals interactions are shown in blue, hydrogen bonds and water bridge are shown in red, π-stacking interactions are shown in green.

This repositioning enables a van der Waals interaction with Pro201 and a hydrogen bond between the adenine moiety and Ser135 (Figure 2C). This distinct position of pevonistat in the hCA XII mimic relative to hCA II provides a structural rationale for the higher inhibitory potency and selectivity of pevonedistat toward the tumor-associated isoform hCA XII. In summary, pevonedistat, classically recognized as first-in-class inhibitor of NAE, also displays a previously underappreciated and isoform selective inhibitory profile against CA isoforms. Kinetic analyses demonstrated preferential inhibition of the tumor-associated isoforms hCA IX and XII. X-ray crystallographic studies provided a structural rationale for this selectivity, revealing how active-site residue differences modulate the positioning of the inhibitor and govern productive hydrophobic, π-stacking, and water bridge interactions. These findings not only explain the extensive whole-blood partitioning of pevonedistat through interaction with erythrocyte CAs but also suggest that CA inhibition may contribute to its antitumor activity in hypoxic tumors where hCA IX and XII are overexpressed. Collectively, our study reveals a dual mechanistic dimension for pevonedistat, linking neddylation inhibition with selective targeting of tumor-associated CAs, and provides a structural framework for exploiting this synergy in anticancer drug design.

## Supplementary Material



## Notes

Experimental sections, supplementary figures and X-ray data collections (PDF)

## Abbreviations

NEDD8: neural precursor cell expressed developmentally downregulated 8
NAE NEDD8: activating enzyme
UBA3: Ubiquitin-like modifier-activating enzyme 3
UPS: ubiquitin-proteasome system
CRL: Cullin-RING ubiquitin ligase
CA: Carbonic Anhydrase
CAI: Carbonic Anhydrase Inhibitors
AAZ: acetazolamide
