# Supplementary material for: Beyond Neddylation Inhibition: X‑ray Structures Reveal Carbonic Anhydrase Isoform Selectivity of Pevonedistat
Source: ACS Med Chem Lett. 2026 May 27;17(6):1393–7. doi: 10.1021/acsmedchemlett.6c00197 (PMC13266636; doi:10.1021/acsmedchemlett.6c00197)
Supplement: Supplementary file 1 [file ml6c00197_si_001.pdf]

Supplementary Material for

Beyond Neddylation Inhibition: X-ray Structures Reveal Carbonic Anhydrase Isoform Selectivity of Pevonedistat

Chiara Baroni[a], Marta Ferraroni[a], Claudiu T. Supuran[b], Andrea Angeli[b]*

[a] Department of Chemistry "Ugo Schiff", University of Florence, Via della Lastruccia 3-13, I-50019, Sesto Fiorentino, Italy

[b] NEUROFARBA Department, Sezione di Scienze Farmaceutiche, University of Florence, Via Ugo Schiff 6, 50019, Sesto Fiorentino, Florence, Italy.

Index

Chemicals *S2*

Evaluation of CA-inhibitory activity *S2*

Crystallization and X-ray data collection *S3*

Summary of Data Collection and Atomic Model Refinement Statistics for hCAs *S4*

Figure S1-2 *S5*

References *S7*

Chemicals

Pevonedistat (MLN4924) (purity ≥95%; Item No. 26150) was supplied by Cayman-Chemical, Michigan, USA. Acetazolamide and topiramate (purity ≥99%) weas supplied by Sigma–Aldrich, Milan, Italy. All other reagents were of analytical grade.

Evaluation of CA-inhibitory activity

An Applied Photophysics stopped-flow instrument was used to assay the CA-catalysed CO_2-hydration activity[1]. Phenol red (at a concentration of 0.2 mM) was used as an indicator, working at an absorbance maximum of 557 nm, with 20 mM HEPES pH 7.5 as buffer and 20 mM sodium sulfate (to maintain a constant ionic strength), following the initial rates of the CA-catalyzed CO_2-hydration reaction for a period of 10–100 s. The CO_2 concentrations ranged from 1.7 to 17 mM for determination of the kinetic parameters and inhibition constants[2]. For each inhibitor, at least six traces of the initial 5–10% of the reaction were used to determine the initial velocity. The uncatalyzed rates were determined in the same manner and were subtracted from the total observed rates. Stock solutions of inhibitor (0.1 mM) were prepared in distilled deionized water and dilutions of up to 0.01 nM were made in the assay buffer. Inhibitor and enzyme solutions were pre-incubated together for 15 min at room temperature prior to the assay in order to allow formation of the enzyme–inhibitor complex. The inhibition constants were obtained by nonlinear least-squares methods using Prism 3 and the Cheng–Prusoff equation, as reported previously, and represent the mean from at least three different determinations. All CA isoforms were recombinant isoforms obtained in-house, as reported previously[3-5].

Crystallization and X-ray data collection

Crystals of hCA I, II and XII mimic were obtained using the hanging drop vapor diffusion method using 24 well Linbro plate. 2 µl of 10 mg/ml solution of hCA II in Tris-HCl 20 mM pH 8.0 were mixed with 2 µl of a solution of 1.5 M sodium citrate, 0.1 M Tris pH 8.0; crystals of hCA XII mimic were obtained mixing 2 µl of 10 mg/ml solution of hCA XII mimic in Tris-HCl 20 mM pH 8.0 with 2 µl of a solution of 2.6 M ammonium sulfate, 0.1 M Tris pH 8.0; crystals of hCA I (10 mg/mL solution of hCA I in in Tris-HCl 20 mM pH 9.0) were obtained mixing 2 µL of protein with 2 µL of a solution of 28% PEG4000, 0.2 M sodium acetate, 0.1 M Tris pH 9.0 and were equilibrated against the same solution at 296 K. The complexes were prepared by soaking the native crystals in the mother liquor solution containing the inhibitor at concentration of 10 mM for one day. The crystals were flash-frozen at 100K using a solution obtained by adding 15% (v/v) glycerol to the mother liquor solution as cryoprotectant. Data on crystals were collected using synchrotron radiation at the XRD2 beamline at Elettra Synchrotron (Trieste, Italy) with a wavelength of 1.000 Å and a DECTRIS Pilatus 6M detector. Data were integrated and scaled using the program XDS[6].

Structure determination

The crystal structures of hCA I (PDB accession code: 3LXE), hCA II (PDB accession code: 4FIK), hCA XII (mimic PDB accession code: 5G0C) without solvent molecules and other heteroatoms was used to obtain initial phases using Refmac5[7]. 5% of the unique reflections were selected randomly and excluded from the refinement data set for the purpose of Rfree calculations. The initial |Fo - Fc| difference electron density maps unambiguously showed the inhibitor molecules. The inhibitor was introduced in the model with 0.5 occupancy for each conformation. Refinements proceeded using normal protocols of positional, isotropic atomic displacement parameters alternating with manual building of the models using COOT[8]. The quality of the final models was assessed with COOT and RAMPAGE[9]. Atomic coordinate was deposited in the Protein Data Bank (PDB accession code: 29UD, 29UH, 29UX). Graphical representations were generated with Chimera[10].

Table S1. Summary of Data Collection and Atomic Model Refinement Statistics for hCAs

	hCA I + Pevonedistat	hCA II + Pevonedistat	hCA XII + Pevonedistat
PDB ID	29UD	29UH	29UX
Wavelength (Å)	1.000	1.000	1.000
Space Group	$P2_12_12_1$	P21	P21
Unit cell (a, b, c, α, β, γ)(Å,°)	61.95, 70.85, 121.54<br>90.00, 90.00, 90.00	42.45, 41.54, 72.53<br>90.00, 104.59, 90.00	42.10, 41.34, 71.60<br>90.00, 103.73, 90.00
Limiting resolution (Å)	1.24-61.21 (1.24-1.27)	1.60-41.54 (1.60-1.63)	1.55-40.90 (1.55-1.58)
Unique reflections	139301 (9139)	28711 (1487)	31629 (1703)
Rmerge (%)	6.4 (279.5)	5.4 (28.1)	7.3 (74.6)
Rmeas (%)	6.7 (296.5)	6.7 (34.9)	9.5 (96.5)
Redundancy	11.6 (8.7)	4.8 (5.1)	3.6 (3.5)
Completeness overall(%)	91.7 (82.3)	88.4 (94.2)	90.3 (98.7)
<I/σ(I)>	17.07 (0.76)	15.8 (4.7)	10.2 (1.6)
CC (1/2)	1.000 (0.361)	0.998 (0.930)	0.997 (0.579)
**Refinement statistics**			
Resolution range(Å)	1.24-61.21	1.60-41.54	1.55-40.90
Rfactor (%)	14.01	16.80	17.32
Rfree(%)	17.04	19.46	20.92
r.m.s.d. bonds(Å)	0.0104	0.0090	0.0082
r.m.s.d. angles (°)	1.8072	1.8289	1.7309
**Ramachandran statistics** (%)			
Most favored	97.3	97.3	96.9
additionally allowed	2.7	2.7	3.1
outlier regions	0.0	0.0	0.0
**Average B factor ($Å^2$)**			
All atoms	22.909	17.330	18.297
Inhibitors	22.795	27.734	59.932
Solvent	34.410	24.953	25.497

Figure S1:

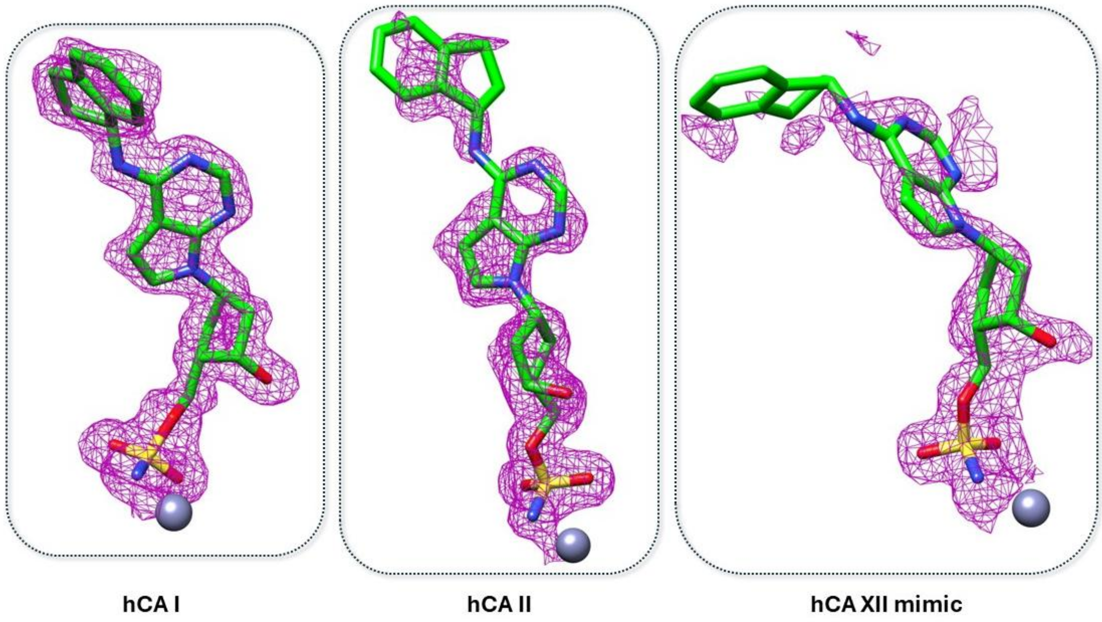

Figure S1. Electron density of inhibitor pevonedistat bound to zinc (grey) in hCA I, hCA II and hCA XII mimic active sites. $2F_o$-F_c maps and contoured to the 1.0 σ level.

Figure S2:

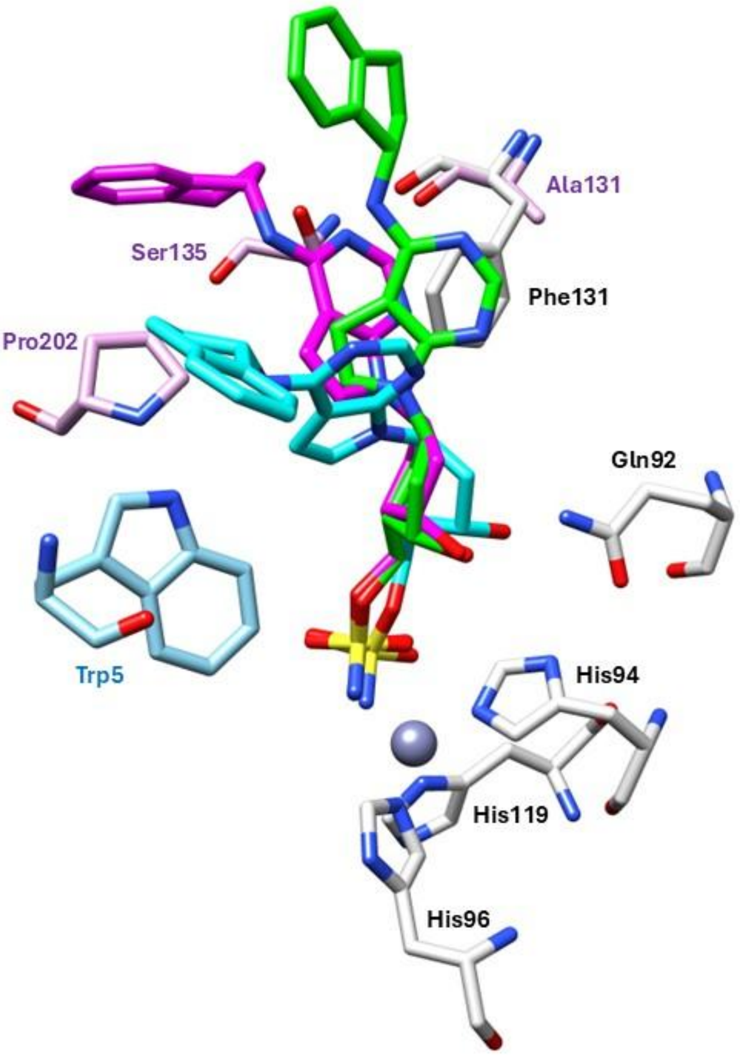

Figure S2. Overlay of pevonedistat with hCA I (cyan), II (green) and XII mimic (magenta). Specific residues are labelled.

References

1. Khalifah, R.G. The carbon dioxide hydration activity of carbonic anhydrase. I. Stop flow kinetic studies on the native human isoenzymes B and C. *J. Biol. Chem*. **1971**, 246, 2561-2573.
2. Supuran CT. Carbonic anhydrases: novel therapeutic applications for inhibitors and activators. *Nat Rev Drug Discov.* **2008**, 7, 168-181.
3. Tanini D, Capperucci A, Ferraroni M, Carta F, Angeli A, Supuran CT. Direct and straightforward access to substituted alkyl selenols as novel carbonic anhydrase inhibitors. *Eur J Med Chem*. **2020**, 185, 111811.
4. Angeli A, Vaiano F, Mari F, Bertol E, Supuran CT. Psychoactive substances belonging to the amphetamine class potently activate brain carbonic anhydrase isoforms VA, VB, VII, and XII. *J Enzyme Inhib Med Chem*. **2017**, 32, 1253-1259.
5. Angeli A, Tanini D, Capperucci A, Malevolti G, Turco F, Ferraroni M, Supuran CT. Synthesis of different thio-scaffolds bearing sulfonamide with subnanomolar carbonic anhydrase II and IX inhibitory properties and X-ray investigations for their inhibitory mechanism. *Bioorg Chem.* **2018**, 81, 642-648.
6. Leslie, A.G.W., Powell, H.R. Procesing diffraction data with mosflm. In: Read RJ, Sussman JL (eds) Evolving methods for macromolecular crystallography, vol 245, NATO Science series, Springer, Dordrecht, **2007**, pp. 41-51.
7. Murshudov, G.N., Vagin, A.A., Dodson, E.J. Refinement of macromolecular structures by the maximum-likelihood method. *Acta Crystallogr D Biol Crystallogr*. **1997**, 53, 240-255.
8. Emsley, P., Lohkamp, B., Scott, W., Cowtan, K. Features and development of Coot. *Acta Crystallogr D Biol Crystallogr.* **2010**, 66, 486-501.
9. Lovell, S.C., Davis, I.W., Arendall III, W.B., de Bakker, P.I.W., Word, J.M., Prisant, M.G., Richardson, J.S., Richardson, D.C., Structure validation by Cα geometry: ϕ,ψ and Cβ deviation. *Proteins*, **2003**, 50, 437-450.
10. Pettersen, E.F., Goddard, T.D., Huang, C.C., Couch, G.S., Greenblatt, D.M., Meng, E.C., Ferrin, T.E., UCSF Chimera-a visualization system for exploratory research and analysis, *J. Comput. Chem.*, **2004**, 25, 1605-1612.
